# Clinical trials comparing norepinephrine with vasopressin in patients with septic shock: a meta-analysis

**DOI:** 10.1186/2054-9369-1-6

**Published:** 2014-05-01

**Authors:** Fei-Hu Zhou, Qing Song

**Affiliations:** Department of Critical Care Medicine, General Hospital of Chinese PLA, Beijing, 100853 China

**Keywords:** Norepinephrine, Vasopressin, Sepsis, Shock, Meta-analysis

## Abstract

**Background:**

The effect of norepinephrine in patients with septic shock remains controversial. We conducted a meta-analysis to compare the mortality rates and benefits of norepinephrine and vasopressin.

**Methods:**

PubMed, EMBASE, and the Cochrane Library database were searched from database inception to December 2013. We selected randomized controlled trials in adults with septic shock and compared norepinephrine with vasopressin. After assessing the heterogeneity of treatment effects across trials using the *I*^2^ statistic, we used a fixed effects model (*P* ≥ 0.1) and expressed the results as risk ratios (RRs) for dichotomous outcomes or as standardized mean differences (SMDs) for continuous data with 95% confidence intervals (CIs). Meta-analysis was conducted using Review Manager 5.1 software.

**Results:**

Seven trials (n = 2323) met the inclusion criteria. Overall, the mortality rate in these seven trials was 36.2% (840/2323). There was no difference in mortality following the use of norepinephrine or vasopressin (RR 1.07; 95%CI 0.97-1.20; *P* = 0.19). Compared to norepinephrine, vasopressin had no significant effect on heart rate (HR) (SMD 0.21; 95%CI −0.08-0.50; *P* = 0.15), mean arterial pressure (MAP) (SMD 0.15; 95%CI −0.15-0.44; *P* = 0.33), cardiac index (CI) (SMD −0.10; 95%CI −0.64-0.44; *P* = 0.73), systemic vascular resistance index (SVRI) (SMD 0.15; 95%CI −0.39-0.70; *P* = 0.58), oxygen delivery (DO_2_) (SMD −0.06; 95%CI −0.62-0.49; *P* = 0.82), oxygen consumption (VO_2_) (SMD 0.03; 95%CI −0.52-0.59; *P* = 0.91) or lactic acid (SMD 0.07; 95%CI −0.23-0.36; *P* = 0.66). No significant heterogeneity was found in these comparisons (*P* ≥ 0.1).

**Conclusions:**

There is not sufficient evidence to prove conclusively that norepinephrine is superior to vasopressin in terms of mortality and hemodynamics. The effects of norepinephrine and vasopressin on patients with septic shock require further study in large randomized controlled trials.

## Background

Septic shock is one of the most challenging medical problems, and severe sepsis accounts for 20% of all admissions to intensive care units (ICUs), including 750,000 cases annually in the United States, with a mortality rate ranging from 28% to 50% [1, 2]. The initial goal-directed resuscitation for septic shock typically includes the administration of intravenous fluids and vasopressors. Although norepinephrine is commonly used and is the recommended agent for the treatment of hypotension in volume-resuscitated hyperdynamic septic shock [3], the effect of norepinephrine on patient-relevant outcomes remains controversial. Recent evidence from a large-scale study revealed that there was no significant difference in the mortality rate between patients with septic shock who were treated with dopamine as the first-line vasopressor agent and those who were treated with norepinephrine [4].

Vasopressin is an endogenously released hormone that has recently emerged as an adjunct to catecholamines for patients with septic shock requiring vasopressor support [5]. When compared with norepinephrine, a study has shown that vasopressin treatment in septic shock is associated with a significant reduction in heart rate but no change in cardiac output or other measures of perfusion [6]. Daley et al. revealed that vasopressin was not inferior to norepinephrine for the achievement of a mean arterial pressure (MAP) goal within the first 6 hours following the onset of septic shock [7]. In another study, Russell et al. demonstrated that low-dose vasopressin did not reduce mortality rates when compared with norepinephrine among patients with septic shock who were treated with catecholamine vasopressors [8].

Due to the continuing controversy regarding whether norepinephrine is superior to vasopressin, we performed a meta-analysis to attempt to determine whether norepinephrine is more effective than vasopressin in reducing overall mortality and improving hemodynamics in septic shock.

## Methods

We performed this meta-analysis following the recommendations of the Preferred Reporting Items for Systematic Reviews and Meta-Analyses (PRISMA statement) guidelines [9].

### Eligibility criteria and information sources

We searched for literature in the PubMed (US National Library of Medicine, Bethesda, MD, USA), EMBASE and Cochrane Library databases from database inception to December 2013. The article types were primarily limited to randomized controlled trials (RCTs) that included patients aged older than 18 years. We also scanned the bibliographies of all relevant studies and recent review articles to identify additional citations.

### Search strategy

We used medical subject heading (MeSH) terms and text words with a Boolean strategy. Cross-searching was performed based on the following 2 categories: (1) different vasopressors (“norepinephrine” OR “vasopressin”); (2) disease (“sepsis” OR “infection” OR “septic shock” OR “shock” OR “systemic inflammatory response syndrome” OR “SIRS”). The limits placed on the literature searches were “human” and “English”.

### Study selection

The study selection was performed by two independent investigators (F.Z. and Q.S.). Studies that compared mortality between norepinephrine and vasopressin use in patients (aged ≥18 years) with septic shock were evaluated and included.

### Data extraction

Raw data were extracted using a standard form for each study, which included the study design, year of publication, total number of patients, and patient characteristics. The main endpoint was 28-day mortality. If mortality was assessed at several time points or only at an undetermined time point in a study, we used data from the last follow-up or the only undetermined time point.

### Quality assessment

The quality of each study included in the meta-analysis was assessed using the Jadad score [10], including the proper conduct of randomization, concealment of treatment allocation, similarity of treatment groups at baseline, clinician blinding, and the description of withdrawals and dropouts.

### Statistical analysis

Statistical analyses were performed using Review Manager, version 5.1 (RevMan, The Cochrane Collaboration, Oxford, the United Kingdom). After assessing for the heterogeneity of treatment effects across trials using the *I*^2^ statistic [11], we used a fixed effects model (*P* ≥ 0.1). The results were expressed as risk ratios (RRs) for dichotomous outcomes or standardized mean differences (SMDs) for continuous data with 95% confidence intervals (CIs), and *P* < 0.05 was considered significant. Publication bias was assessed using funnel plots.

## Results

### Study selection

**Figure 1:**
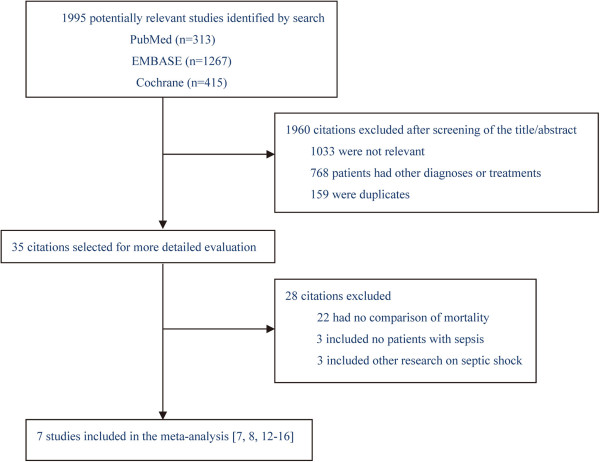
**Quorum chart of the study cohort.** The search was conducted using the PubMed, EMBASE, and Cochrane Library databases from database inception to December 2013.

A total of 1995 studies were identified. We retrieved 35 articles for detailed evaluation, of which 28 were excluded (Figure 1). Seven trials (2323 patients) met the criteria for inclusion [7, 8, 12–16]. All studies compared the effects of norepinephrine and vasopressin in patients with septic shock using a primary outcome such as survival, hemodynamics, or acute physiology and chronic health evaluation (APACHE) II score (Table 1).

**Table 1:** **Characteristics of the included trials**

Source	Number of patients	Mean age (years)	Male (%)	Center	Mean APACHE II/SAPS II/SOFA score	Blood pressure (mmHg)
Russell JA, 2013 [12]	394	62.8	233 (59.1)	M	26.8/NR/NR	MAP <65
Daley MJ, 2013 [7]	130	58.5	69 (53.1)	S	27.8/NR/NR	MAP <65
Gordon AC, 2010 [13]	778	61.8	475 (61.0)	M	27.1/NR/NR	MAP 72.7 (NE maintenance)
Russell JA, 2009 [14]	190	61	116 (61.1)	M	26.5/NR/NR	MAP <60
Morelli A, 2009 [15]	45	65.7	33 (73.3)	S	NR/60/NR	MAP <65
Russell JA, 2008 [8]	778	60.6	475 (61.1)	M	27.1/NR/NR	MAP 72.5 (vasopressor maintenance)
Lauzier F, 2006 [16]	23	54.7	14 (60.9)	M	23.2/NR/8.9	MAP <60

APACHE, acute physiology and chronic health evaluation; SAPS, simplified acute physiology score; SOFA, sequential organ failure assessment; MAP, mean arterial pressure; S, single-center trial; M, multicenter trial; NE, norepinephrine; NR, not reported.

### Study characteristics

Five multicenter studies [8, 12–14, 16] and two single-center studies [7, 15] were identified. The characteristics of the included trials are shown in Table 1. These trials were reported between 2006 and 2013, and the mean age of the study participants ranged between 54.7 and 62.8 years. The proportion of men ranged from 53.1% to 61.1%. The mean APACHE II score was between 23.2 and 27.8. All patients with sepsis or septic shock were diagnosed according to the American College of Chest Physicians/Society of Critical Care Medicine Consensus Conference criteria [17].

### Risk of bias within studies

Six of the citations included [8, 12–16] were randomized controlled trials, and one was a cohort study [7]. Blinding was performed in four studies [8, 12–14]. The mean Jadad score of the six randomized controlled trials was 4 (Table 2).

**Table 2:** **Quality assessment of the six randomized controlled trials included in the meta-analysis**

Source	Randomization	Allocation concealment	Blinding	Description of withdrawals and dropouts	Jadad score
Russell JA, 2013 [12]	Yes	Uncertain	Yes	Yes	3
Gordon AC, 2010 [13]	Yes	Adequate	Yes	Yes	5
Russell JA, 2009 [14]	Yes	Adequate	Yes	Yes	5
Morelli A, 2009 [15]	Yes	Adequate	Uncertain	Yes	3
Russell JA, 2008 [8]	Yes	Adequate	Yes	Yes	5
Lauzier F, 2006 [16]	Yes	Adequate	Uncertain	Yes	3

### Effect of norepinephrine versus vasopressin on mortality

**Figure 2:**
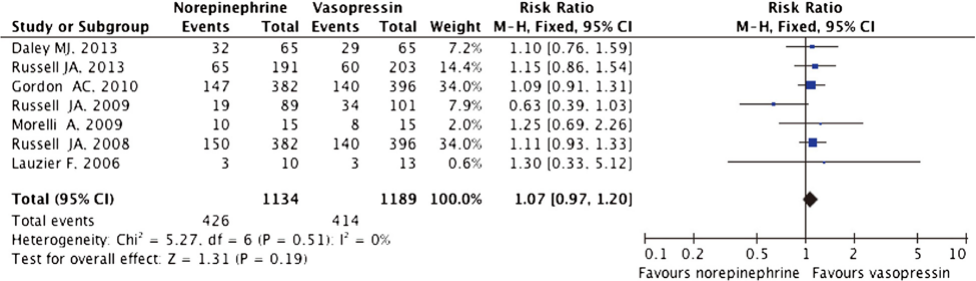
**Risk ratio of mortality for norepinephrine versus vasopressin.** Pooled risk ratios (RRs) were obtained using a fixed effects model; CI indicates the confidence interval; The size of the data markers indicates the weight of the study.

The mortality rate in the seven trials was 36.2% (840/2323). No difference in mortality was identified when comparing norepinephrine and vasopressin (RR 1.07; 95%CI 0.97-1.20; P = 0.19). No significant heterogeneity was found in this comparison (*I*^2^ = 0%, P = 0.51) and the fixed effects model was used (Figure 2). Because one trial [7] was a cohort study, we also performed a meta-analysis of the other six trials [8, 12–16]. Similarly, no difference in mortality was found when comparing these two groups (RR 1.07; 95%CI 0.96-1.20; P = 0.22; *I*^2^ = 5%, P = 0.39).

### Effect of norepinephrine versus vasopressin on hemodynamic and metabolic parameters

**Figure 3:**
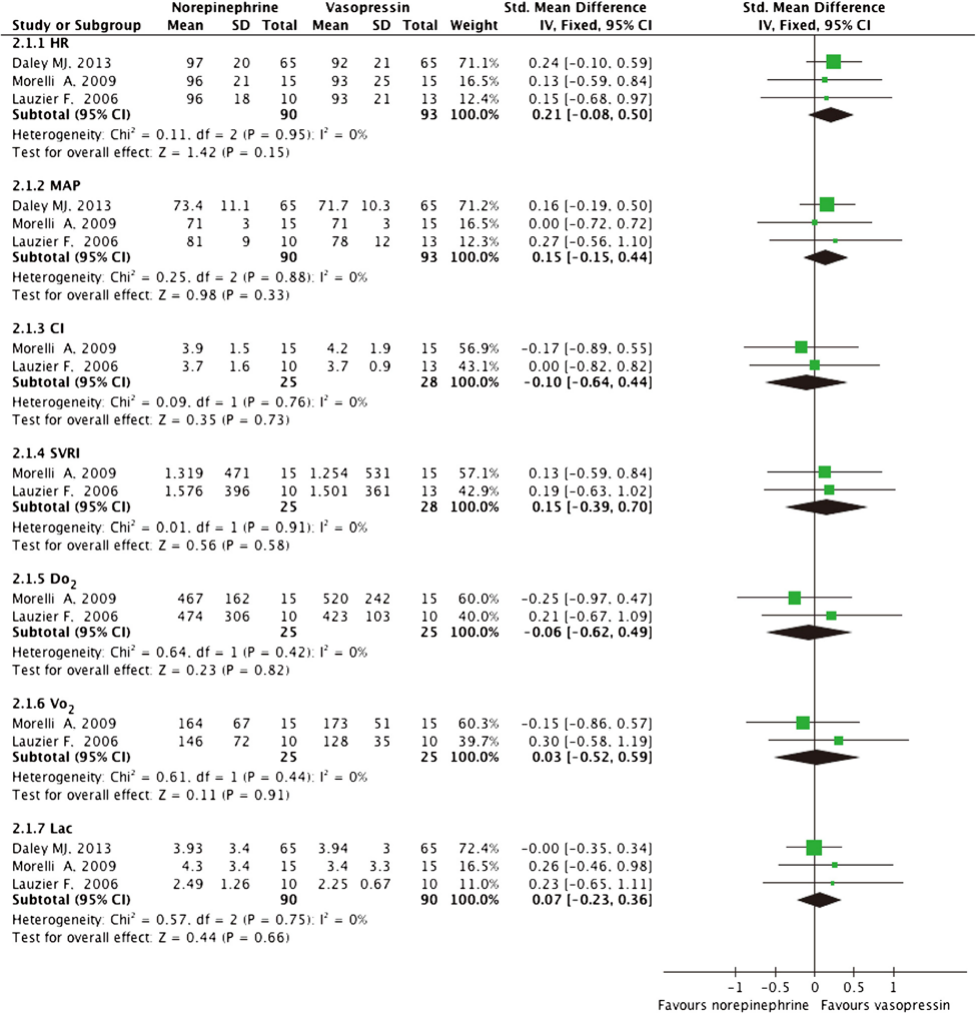
**Effect of norepinephrine versus vasopressin on hemodynamic and metabolic parameters.** HR, heart rate; MAP, mean arterial pressure; CI, cardiac index; SVRI, systemic vascular resistance index; DO_2_, oxygen delivery; VO_2_, oxygen consumption; MPAP, mean pulmonary arterial pressure; SMD, standardized mean difference; CI, confidence interval; IV, inverse variance method.

Compared to norepinephrine, vasopressin had no significant effect on heart rate (HR) (SMD 0.21; 95%CI −0.08-0.50; *P* = 0.15), MAP (SMD 0.15; 95%CI −0.15-0.44; *P* = 0.33), cardiac index (CI) (SMD −0.10; 95%CI −0.64-0.44; *P* = 0.73), systemic vascular resistance index (SVRI) (SMD 0.15; 95%CI −0.39-0.70; *P* = 0.58), oxygen delivery (DO_2_) (SMD −0.06; 95%CI −0.62-0.49; *P* = 0.82), oxygen consumption (VO_2_) (SMD 0.03; 95%CI −0.52-0.59; *P* = 0.91) or lactic acid (SMD, 0.07; 95%CI −0.23-0.36; *P* = 0.66). No significant heterogeneity was found in these comparisons (*P* ≥ 0.1, Figure 3).

### Publication bias analyses

**Figure 4:**
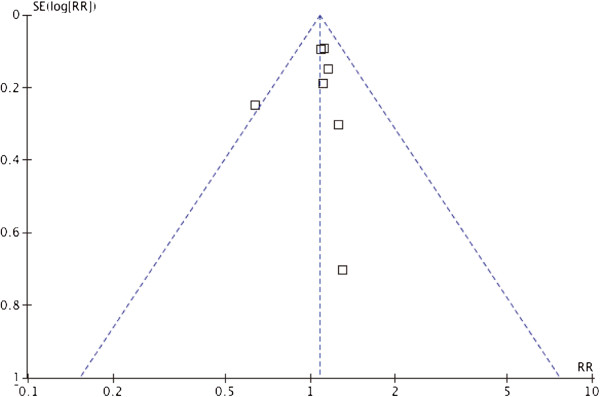
**Assessment of publication bias using a funnel plot.**

Publication bias was evaluated using a funnel plot, and the primary comparisons of mortality are presented. The funnel plots of this primary outcome did not suggest major asymmetry, indicating no significant publication bias (Figure 4).

## Discussion

Seven trials including 2323 patients with septic shock that compared the use of norepinephrine to vasopressin were identified and included in this review. The main results revealed that the survival of patients treated with norepinephrine was not significantly different from those treated with vasopressin. Furthermore, there was also no evidence indicating that norepinephrine is superior to vasopressin in improving hemodynamics.

Vasopressors should be initiated in patients with septic shock if fluid resuscitation fails to restore adequate arterial pressure and organ perfusion, and the effects of vasopressors differ based on the targeted adrenergic receptors, resulting in heterogeneity of their physiological effects [18]. Although both dopamine and norepinephrine are recommended as first-line vasopressor agents in the treatment of septic shock [3], vasopressin, which is a peptide hormone released by the pituitary in response to decreased intravascular volume, has been used in patients with septic shock [18, 19]. In a multi-center double-blind randomized controlled trial of vasopressin versus norepinephrine in adult patients who had septic shock, Gordon et al. revealed that patients with septic shock who were at risk of kidney injury had reduced progression to renal failure and reduced 28-day mortality when treated with vasopressin in comparison to those treated with norepinephrine [13]. However, our meta-analysis did not find a significant difference in mortality between norepinephrine and vasopressin, which was consistent with more recent randomized clinical trials [8, 15, 16]. It is likely that no single pressor has been definitively shown to have a mortality benefit over another in patients with septic shock. It is possible that a continuous infusion of low-dose vasopressin, when given as first-line vasopressor agent in septic shock, is effective in reversing sepsis-induced arterial hypotension and reducing norepinephrine requirements.

For septic patients, once the inflammatory response has been induced, a marked decrease in the SVRI results from arterial and venous dilation, which is accompanied by leakage of plasma into the extravascular space, leading to relative hypovolemia [20]. Recent randomized clinical trials demonstrated that survivors of septic shock had greater decreases in cytokines, chemokines and growth factors in early septic shock. Furthermore, vasopressin decreased 24-hour plasma cytokine levels more than did norepinephrine [12]. In the present study, we compared norepinephrine to vasopressin and found no significant differences in HR, MAP, CI, or SVRI. This was not consistent with a previous trial [16], in which vasopressin was reported to increase the SVRI and decrease the CI when compared with baseline, whereas norepinephrine did not [16]. The hemodynamic impact of norepinephrine on the treatment of septic shock compared to vasopressin, however, requires further evaluation in randomized clinical trials.

For patients with septic shock, it is imperative to restore adequate perfusion pressure and oxygen delivery. It is evident that inadequate systemic hemodynamics, i.e., systemic DO_2_ and VO_2_, can impair splanchnic blood flow and oxygenation [21]. Although increased renal circulation and splanchnic blood flow have been reported in cases of hyperdynamic septic shock treated with norepinephrine [22–24], no significant differences in DO_2_, VO_2_ or lactate were found between norepinephrine and vasopressin in our meta-analysis. Because one of the rationales for catecholamine administration in septic patients is to increase DO_2_ due to the relationship between DO_2_ and VO_2_[25], norepinephrine may therefore be questionable as a preferential treatment when compared with vasopressin in this context.

There are some limitations to this meta-analysis. First, although the mean Jadad score of the included trials was 4, indicating that most of the trials were of high quality, one cohort study was included in this meta-analysis, which may limit the strength of the analysis. Second, although seven trials were included in the analysis, the actual sample size for specific comparisons in subgroup analyses was small, and publication bias was only evaluated using a funnel plot with seven studies, which may have affected the findings. The effects of norepinephrine and vasopressin in patients with septic shock require further evaluation in large-scale randomized controlled trials.

## Conclusions

In conclusion, pooled results of seven trials show that there is not sufficient evidence to prove conclusively that norepinephrine is superior to vasopressin in terms of mortality and hemodynamics. The effects of norepinephrine and vasopressin on patients with septic shock require further study in large randomized controlled trials.

## Abbreviations

ICU: Intensive care unit
RCTs: Randomized controlled trials
MeSH: Medical subject heading
CI: Confidence interval
HR: Heart rate
MAP: Mean arterial pressure
CI: Cardiac index
SVRI: Systemic vascular resistance index
MPAP: Mean pulmonary arterial pressure
SMD: Standardized mean difference
IV: Inverse variance.
